# Straylight of Explanted Silicone Oil Samples to Predict Emulsification

**DOI:** 10.1016/j.xops.2024.100558

**Published:** 2024-05-23

**Authors:** Maximilian Hammer, Leoni Britz, Sonja Schickhardt, Donald Munro, Ramin Khoramnia, Alexander Scheuerle, Christian S. Mayer, Philipp Uhl, Grzegorz Łabuz, Gerd Uwe Auffarth

**Affiliations:** 1 Department of Ophthalmology, University Clinic Heidelberg, Heidelberg, Germany; 2 David J Apple Laboratory for Vision Research, Heidelberg, Germany; 3 Ortenau-Klinikum, Department of Ophthalmology, Offenburg-Kehl, Germany; 4 Heidelberg University, Institute for Pharmacy and Molecular Biotechnology, Heidelberg, Germany

**Keywords:** Vitreoretinal surgery, Silicone oil, Forward light scattering, Emulsification, Endotamponade

Although silicone oil tamponades have been greatly improved on during the last decades,^1^ 1 disadvantage of silicone oil endotamponades is their tendency to emulsify.^2^ In a recent study by Al-Dwairi et al,^3^ other parameters, such as the contact angle, chemical bonding, and light transmittance, were identified as altered after using silicone oil as an endotamponade in 5 samples of 5 patients. Forward light scattering is another parameter likely to be altered by the water-in-oil droplets: one which can be measured time-efficiently in clinical practice. This approach could prove interesting for detection of early emulsification in the eye. Thus, we investigated straylight of Siluron 5000 (Fluoron GmbH) after use as an intraocular tamponade and correlated the results with clinical data.

**Figure 1:**
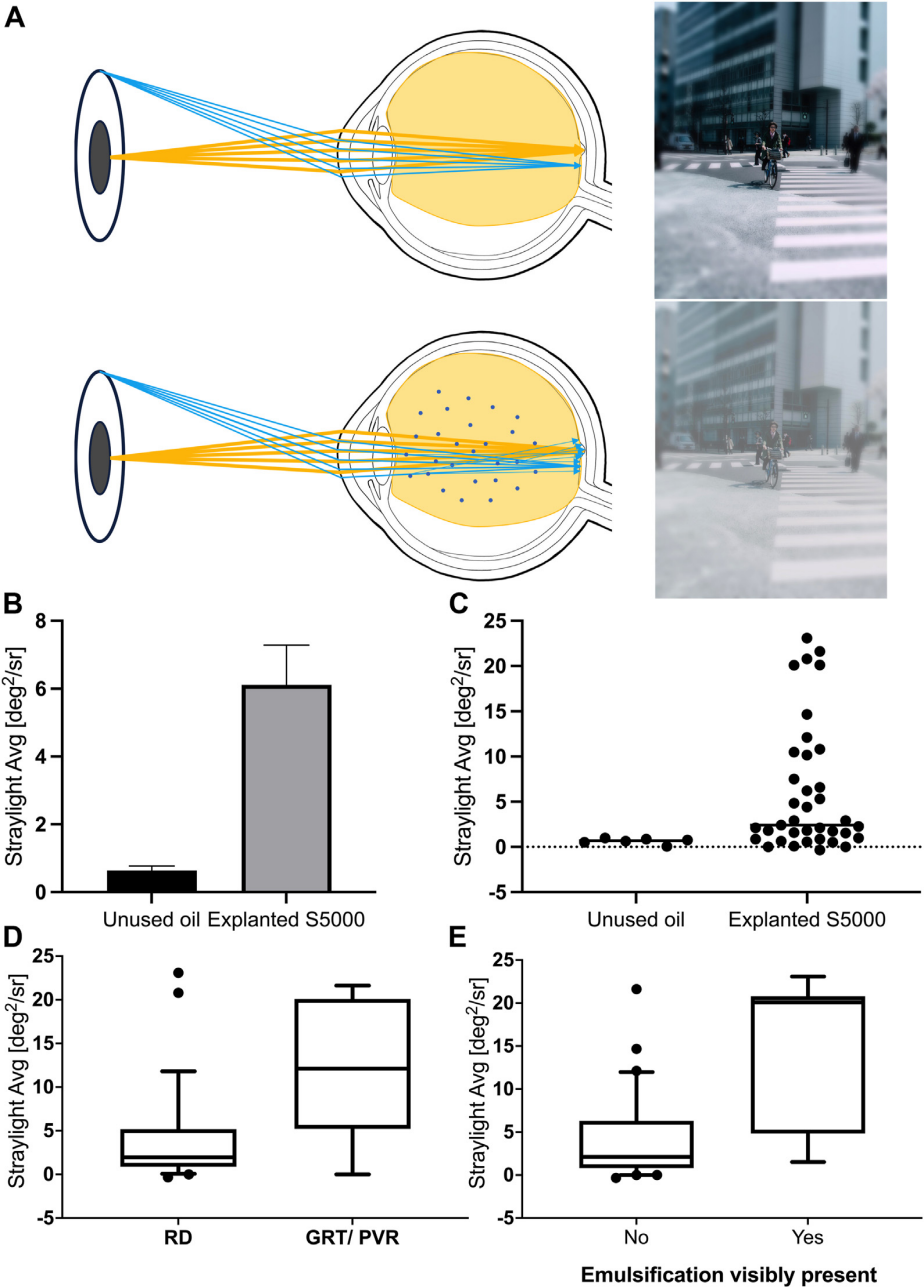
**A,** In a silicone oil-filled eye, water-in-oil-emulsions can occur (bottom). In comparison to a situation where no straylight is induced by emulsification droplets (top), rays of light can be scattered in a forward direction onto the retina. This can cause, e.g., foggy vision as seen on the respective picture. To be noted, all measurements in this study were conducted in vitro. **B, C,** The straylight of explanted silicone oil samples showed no normal distribution. Most samples showed only little to no increase in straylight; on the other hand, a subgroup showed greatly increased straylight values. Minor negative values arise from the oil reaching lower straylight values than baseline measurements of balanced salt solution used as a comparison. **D,** Cases receiving silicone oil endotamponade for giant retinal tears (GRTs, a severe subform of retinal detachment [RD]) or proliferative vitreoretinopathy (PVR) showed higher straylight values compared with cases with a primary rhegmatogenous RD. Retinal detachment: 28 cases; GRT/PVR: 9 cases. **E,** Samples from patients with visible emulsification induced a greater amount of straylight.

Silicone oil from 37 eyes of 37 patients was used. Only silicone oil was aspirated in a first syringe to minimize interference with the balanced salt solution infusion. This study was conducted in accordance with Good Clinical Practice guidelines and the Declaration of Helsinki. Because all tested samples were included in the Apple Laboratory explant registry, no informed consent was necessary. Institutional review board approval was granted by the Heidelberg University ethics committee (DRKS00007837). To measure straylight, we used a methodology adapted by Łabuz et al^4^ for in vitro evaluation of forward light scattering by using a custom mount on the C-Quant (Oculus GmbH) device (Fig 1A). Patients’ information was screened for factors associated with silicone oil emulsification. Stata 17BE and Prism 8 were used for statistical analysis. *T* tests or Mann–Whitney *U* tests were conducted as appropriate.

Silicone oil removal was uneventful in all cases. Explanted samples showed a significantly higher straylight compared with unused control oils (*P* < 0.001). Interestingly, the straylight of silicone oil samples was not normally distributed; only a subset of around one third of samples showed a greater increase in straylight (Fig 1B, C). The duration of the tamponade was not significantly correlated with straylight (*r*^2^ = 0.08, *P* = 0.09). The samples explanted from 9 patients that received the oil tamponade for a tractive detachment due to proliferative vitreoretinopathy (PVR) or for giant retinal tears, a severe subtype of a rhegmatogenous retinal detachment, showed a significantly greater straylight compared with oil removed from patients that were treated for uncomplicated rhegmatogenous retinal detachments (*P* < 0.01, 28 cases; Fig 1D). We further evaluated retinotomies as a possible factor to increase silicone oil straylight; no significant correlation was seen (*P* = 0.72). Seven patients received an anterior chamber washout after silicone oil removal because of visible oil droplets. These oil samples showed significantly higher straylight values (*P* < 0.001, Fig 1E). Endolaser was used in most cases at the time of the initial vitrectomy and not associated with higher straylight values (*P* = 0.29). Similarly, in 3 cases, buckling surgery performed during oil insertion or in an earlier surgery was not associated with increased straylight values (*P* = 0.48). Acute intraocular inflammation, a known risk factor for silicone oil emulsification, was only present in 1 of the 37 cases. This case had a remarkably increased level of straylight of 20.8 degrees^2^/sr. Our series included only 1 case with pseudoexfoliation syndrome. It showed a significantly higher straylight value compared with baseline 7.5 degrees^2^/sr. Straylight was not increased in 3 samples extracted from diabetic patients (*P* = 0.61) compared with other explanted samples.

We consider that our results recommend that intraocular straylight may be a marker for early emulsification before it is detectable by slit-lamp examination. The 25% of oil samples with the greatest straylight caused similar straylight to a crystalline lens of a 70-year-old patient of around 10 degrees^2^/sr.^5^ Given the retinal comorbidities warranting the use of silicone oil, such straylight values may significantly worsen the already cautious visual prognosis. Previous studies suggested that early silicone oil emulsification generates silicone oil droplets with a diameter of around 1 μm,^6^ a size which cannot be detected noninvasively with current diagnostic instruments. Visible emulsification indicates that the process has already substantially progressed, and possible complications may have already occurred. Optimally, a noninvasive way to detect early emulsification could enhance the decision making on when to remove silicone oil to prevent further complications such as intraocular pressure spikes or glaucoma. Based on this laboratory data, a clinical study on the dynamics of straylight in patients with silicone oil endotamponade could further illuminate implications of measuring straylight and improving visual outcomes of patients with silicone oil endotamponade.

Data on risk factors for emulsification are inconsistent. We found that PVR and giant retinal tears as indications for surgery lead to higher straylight values at silicone oil removal. Giant retinal tears and PVR are characterized by an ocular inflammation and a possible blood–retinal barrier breakdown, known to lower the interfacial tension between silicone oil and the aqueous.^7^ This could lead to earlier and more severe emulsification, presenting as higher straylight values of explanted samples. Accordingly, in the only case in which strong intraocular inflammation was present at silicone oil removal, greatly increased straylight values were observed.

The tamponade duration was previously established as a risk factor for silicone oil emulsification. In this study, no correlation was apparent. However, the cases underwent timely silicone oil removal most likely influencing this association.

This study has inherent limitations. These are the results from a laboratory study of explanted oil samples, and it remains unclear if the increase in straylight can be quantified in patients during silicone oil endotamponade. The purpose of this study, however, was to evaluate straylight as a statistical proxy for ongoing silicone oil emulsification. Additionally, we are only able to evaluate the straylight of the explanted silicone oil and not the aqueous humor because it is constantly diluted by the infusion of balanced salt solution.

Although most explanted silicone oil samples induced a low amount of straylight, individual samples induced straylight as a mild-to-medium lenticular opacification, especially cases with visible anterior chamber silicone oil droplets. This laboratory study suggests that straylight in the silicone oil-filled eye could be a noninvasive parameter to estimate intraocular silicone oil emulsification. The results of this laboratory study should be translated into a clinical study using the C-Quant device next.
